# Bead-based suspension array for detection and identification of tick-borne Borrelia species

**DOI:** 10.1186/1756-3305-7-S1-P7

**Published:** 2014-04-01

**Authors:** RP Achterberg, CB Van Solt-Smits, KE Hovius, H Sprong, M Fonville, FM De Bree, FJ Van der Wal

**Affiliations:** 1 Infection Biology, Central Veterinary Institute, Lelystad, the Netherlands; 2 Companion Animal Hospital 't Heike, Veldhoven, the Netherlands; 3 Laboratory for Zoonoses and Environmental Microbiology, National Institute of Public Health and Environment (RIVM), Bilthoven, the Netherlands

## 

In the Netherlands, screening for tick-borne pathogens is performed using real time PCR on DNA extracted from ticks, followed by sequencing of positive samples, thereby disallowing identification in case of double-infections. Until recently, testing was performed with several reverse line blots (RLBs), each containing ~10 different probes. Advantages of RLBs are the capabilities to differentiate between bacterial species and to detect double infections, disadvantages are that RLBs are fastidious, elaborate, and time-consuming.

The aim of this work was to 'test drive' bead-based suspension arrays for the rapid detection and identification of tick-borne Borrelia species. The platform used is the flow cytometry-based xMAP technology of Luminex, in which fluorescent nanoparticles (beads) are used to build multiplex assays simply by mixing different bead sets that are covered with specific probes. Each bead set contains a different ratio of red and infrared fluorophores, which enables identification of beads that pick up a third fluorophore by hybridization to a labelled target molecule.

Using standard chemistry, aminated oligonucleotides were covalently linked to carboxylated Luminex beads, resulting in bead sets with general and specific probes for the 23S-5S intergenic spacer (IGS) of Borrelia species. Presence of probes on the beads was verified by hybridization of complementary biotinylated oligonucleotides in presence of a mixture of 1000 beads per bead set, using a 30 minute incubation at 37°C in a NaCl/Tris buffer, followed by labelling with streptavidin-phycoerythrin. Of each mixture, a minumum of 100 beads per bead set were investigated by flow cytometry on a dedicated Luminex instrument.

To evaluate the suspension array, DNA of nearly 1000 ticks was isolated and used to generate amplicons with a universal PCR targeting the 23S-5S IGS of Borrelia spp. All PCR reactions were then used in a direct hybridization assay in presence of a mixture of probe-containing beads. Out of 981 ticks, ^~^20% were found to carry one or more Borrelia species (resp. 15 and 5%). The most prevalent species was B. afzelii, followed by B. burgdorferi sensu stricto, B. garinii, B. sensu lato (unidentified) and B. valaisiana. These results show the viability of bead-based suspension arrays for (relatively) rapid detection and identification of multiple species, but at this stage need verification by a 'gold standard'. Currently, confirmation of these results is being performed by next generation sequencing.

## Funding

this work was partly funded by the European Union Seventh Framework Programme (FP7/2007-2013) under Grant Agreement Number 222633 (WildTech).

